# Intraoperative Ultrasound Guidance in Laparoscopic Adrenalectomy: A Retrospective Analysis of Perioperative Outcomes

**DOI:** 10.3390/diagnostics15070898

**Published:** 2025-04-01

**Authors:** Ionela Mihai, Adrian Boicean, Horatiu Dura, Cosmin Adrian Teodoru, Dan Georgian Bratu, Cristian Ichim, Samuel Bogdan Todor, Nicolae Bacalbasa, Alina Simona Bereanu, Adrian Hașegan

**Affiliations:** 1 Faculty of Medicine, Lucian Blaga University of Sibiu, 550169 Sibiu, Romania; ionela.mihai@ulbsibiu.ro (I.M.); adrian.boicean@ulbsibiu.ro (A.B.); adrian.teodoru@ulbsibiu.ro (C.A.T.); cristian.ichim@ulbsibiu.ro (C.I.); samuelbogdant@gmail.com (S.B.T.); alina.bereanu@ulbsibiu.ro (A.S.B.); adrian.hasegan@ulbsibiu.ro (A.H.); 2 Surgery Department, University of Medicine and Pharmacy “Carol Davila” Bucharest, 020021 Bucharest, Romania; nicolaebacalbasa@gmail.com

**Keywords:** intraoperative ultrasound, adrenalectomy, tumor, laparoscopy, retroperitoneal, transperitoneal, outcomes

## Abstract

**Background**: This study aimed to evaluate the advantages of integrating intraoperative ultrasound (IOUS) into laparoscopic adrenal surgery by assessing its impact on perioperative outcomes and identifying potential complications. **Methods**: This retrospective study analyzed 128 patients with adrenal gland tumors who underwent a laparoscopic adrenalectomy by comparing those who received intraoperative ultrasound guidance with those who did not. The procedures were performed using either the transperitoneal or the lateral retroperitoneal approach. **Results**: The IOUS-guided group had significantly lower blood loss (*p* < 0.001) and a shorter hospitalization duration (*p* = 0.005) compared with the non-IOUS group. No intraoperative complications were observed in the IOUS group, whereas three complications occurred in the non-IOUS group, including peritoneal breaches and minor liver damage. The retroperitoneal approach demonstrated superior perioperative outcomes, with a shorter operative time (*p* < 0.001), reduced blood loss (*p* < 0.001), earlier resumption of oral intake and lower postoperative analgesia requirements (*p* < 0.001). **Conclusions**: Intraoperative ultrasound enhanced the surgical precision in laparoscopic adrenalectomy, which reduced the blood loss, shortened the hospital stays and minimized the intraoperative complications.

## 1. Introduction

Ultrasonography is a fundamental tool in both diagnostic and therapeutic settings, offering essential support in the evaluation of internal organs and superficial tissues [1]. As a non-invasive, rapid imaging modality without ionizing radiation, it remains a first-line investigation for abdomino-pelvic disorders. Technological advancements have expanded its applications, introducing three-dimensional (3D) and four-dimensional (4D) imaging, which may become standard in the near future [2,3]. Specialized intraoperative ultrasound (IOUS) probes now allow for precise anatomical visualization during both conventional and minimally invasive procedures. In open surgery, IOUS can be performed with transcutaneous-compatible probes, while in laparoscopy, dedicated transducers designed for insertion through trocars are required. In 1960, IOUS was first proposed, and by 1961, physicians successfully used A-mode ultrasound to locate kidney stones. In 1962, Hayashi and colleagues demonstrated its utility in detecting gallstones. Despite these early successes, adoption was initially limited due to challenges with image acquisition and interpretation. The development of B-mode ultrasound in the 1970s significantly improved the real-time imaging resolution and clinical applicability [4,5,6,7,8]. In minimally invasive urological surgery, IOUS has become increasingly important for identifying and delineating anatomical and pathological structures. Although preoperative imaging with computed tomography (CT) and magnetic resonance imaging (MRI) provides essential details, IOUS enhances the intraoperative accuracy by enabling real-time tumor localization, guiding biopsies, refining staging, improving hemostasis, and assisting in optimal incision planning and surgical approach selection [9,10,11,12,13].

The refinement of IOUS technology has expanded its application across multiple surgical specialties, including endocrine, cardiac, neurological, vascular, digestive and breast surgeries. The rapid shift toward laparoscopic and robotic procedures, coupled with advances in ultrasonography, has renewed interest in its integration into minimally invasive surgery [6,7,14,15,16,17,18,19].

A laparoscopic adrenalectomy demands exceptional precision due to the complex anatomical location of the adrenal glands. IOUS has been widely used to improve tumor localization, particularly in cases of small adrenal tumors. While it may not be necessary in all cases, it is particularly beneficial when performing partial tumor resections or assessing a tumor extension. In laparoscopic or robotic adrenal surgery, IOUS is invaluable for identifying the adrenal gland in patients with excessive adipose tissue, particularly those with a BMI over 35. It also aids in assessing tumor invasion, detecting distant metastases and visualizing vascular structures as small as 1 mm in diameter. Adrenal adenomas typically appear well-defined, homogeneous, and either hyperechoic or isoechoic compared with normal adrenal tissue. In contrast, adrenal carcinomas often exhibit an inhomogeneous texture with areas of necrosis or intratumoral hemorrhage, with or without extension into adjacent tissues [20,21,22,23,24,25]. Despite the advantages of IOUS, its use in laparoscopic adrenal surgery has been reported in relatively few studies and comprehensive analyses of its impact on perioperative outcomes remain scarce. A major challenge in laparoscopic adrenalectomy is the limited visibility of anatomical landmarks, particularly in patients with complex anatomical variations. Surgeons often rely on tactile feedback and indirect visual cues, which may be insufficient for distinguishing the adrenal gland from the surrounding structures.

This increases the risk of inadvertent injury to adjacent organs, such as the liver, kidney, spleen or major blood vessels. Without real-time IOUS imaging, accurately identifying the correct dissection plane becomes more difficult, potentially prolonging the operative time and increasing blood loss. The absence of IOUS also raises the risk of complications, including hemorrhage or unintentional damage to critical structures, as the surgical team lacks dynamic, on-demand imaging to guide and refine the dissection [26,27,28]. This study aimed to evaluate the impact of IOUS integration in laparoscopic adrenal surgery. We assessed key perioperative outcomes—blood loss, hospitalization duration and complication rates—by comparing procedures performed with and without IOUS guidance. Additionally, we analyzed outcomes between retroperitoneal and transperitoneal approaches to identify the optimal technique for laparoscopic adrenalectomy.

## 2. Materials and Methods

### 2.1. Study Design

This retrospective study analyzed 128 patients with adrenal gland tumors who underwent a laparoscopic adrenalectomy by comparing those who received intraoperative ultrasound (IOUS) guidance with those who did not. The procedures were performed using either the transperitoneal or the lateral retroperitoneal approach. All surgical procedures were performed by the same operating team at the Urology Clinic of the County Clinical Emergency Hospital of Sibiu, Romania, over a five-year period.

### 2.2. Inclusion/Exclusion Criteria

The patients aged 18 years or older and diagnosed with adrenal tumors (primary or secondary) were included if they had undergone biohumoral assessments with hormonal profiling, as well as CT and MRI examinations. A multidisciplinary evaluation was conducted that involved endocrinologists, cardiologists and anesthesiologists. The patients with unclear diagnoses or those without follow-up at our center were excluded. However, patients with severe comorbidities (e.g., renal failure, decompensated cardiovascular disease) and those with a history of prior abdominal surgeries were not excluded.

### 2.3. Surgical Preparation

All the patients provided written informed consent after receiving detailed explanations regarding the treatment options, potential intraoperative and postoperative complications, and the implications of proceeding without surgery. During the preoperative period, treatment strategies were individualized based on the clinical criteria, medical history, tumor location and origin, potential intra- and postoperative risks associated with each surgical approach, recovery time and socio-professional reintegration. The patients with functional tumors received personalized management based on biochemical assessments, which were all supervised by the same surgical team.

### 2.4. Evaluated Outcomes

IOUS was performed in 58 patients to assess its effectiveness and advantages in a laparoscopic adrenalectomy. The primary objectives were to facilitate adrenal gland vascularization mapping, identify vascular anomalies, assess adrenal gland infiltration and surrounding tissue involvement, reduce the operative time, minimize the bleeding risk, and enable more precise and efficient dissection.

### 2.5. Statistical Analysis

Continuous variables were reported as the median (interquartile range) and compared using the Kruskal–Wallis test or the Mann–Whitney U test for dichotomous variables. Categorical variables were expressed as a number (percentage, %), with comparisons performed using the Chi-square or Fisher’s exact test. Normality was assessed using the Shapiro–Wilk test, with a significance level set at *p* < 0.05 (two-tailed). Continuous variables following a normal distribution were analyzed using Student’s *t*-test, while non-normally distributed variables were analyzed using the Mann–Whitney U test. To reduce the selection bias, 1:1 propensity score matching (PSM) with a caliper of 0.2 was applied to balance the IOUS-guided and non-IOUS-guided groups, with adjustments for age, BMI, tumor size and surgical approach.

Additionally, a multivariable linear regression analysis was conducted to determine the independent impact of IOUS guidance on blood loss and hospitalization duration, adjusting for relevant clinical variables. IBM SPSS version 26.0 was used for all the statistical analyses.

### 2.6. Surgical Technique Details

For all the patients, general anesthesia was initiated with orotracheal intubation, followed by the establishment of central venous access via the internal jugular vein. A urethral catheter was placed in all cases, while a nasogastric tube was inserted for the patients that underwent a transperitoneal approach.

In cases where pheochromocytoma was suspected based on clinical and paraclinical evaluations, an epidural catheter placement was additionally performed. This measure significantly improved the hemodynamic control by facilitating both the induction and maintenance of controlled hypotension through its vasodilatory and sympatholytic effects. The lateral retropertional approach involved positioning the patient in the lateral decubitus position, with the main surgeon and assistant positioned behind the patient and the second assistant standing in front of the patient. An incision of about 1.5 cm was made at the tip of the 12th rib to allow for access to the retroperitoneal space.

Access to the retroperitoneum was achieved by blunt dissection of the subcutaneous tissue using scissors, followed by careful penetration of the retroperitoneal fascia. The retroperitoneal space was then expanded using a digital balloon technique.

**Figure 1:**
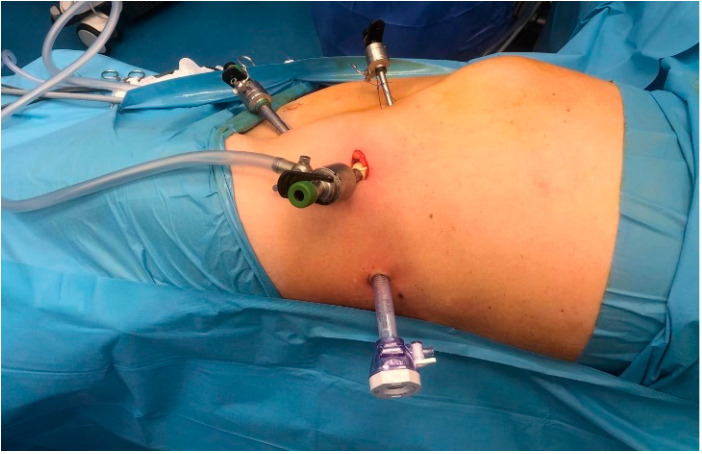
The positioning of the four trocars.

The placement of the four trocars was carried out under digital or visual guidance to ensure careful navigation around key anatomical structures in the retroperitoneal space, including the psoas muscle, ureter, diaphragm and the posterosuperior renal pole (Figure 1).

Once the working space was created, the Gerota fascia at the level of the upper renal pole was incised toward the medial surface, where the central vein of the adrenal gland was identified, ligated with hem-o-lok clips and then divided. To mobilize and extract the adrenal gland into the organ bag, the gland was circumferentially dissected, with electrocoagulation of the surrounding vascular structures. Depending on the size of the adrenal gland, it may be removed either through an enlarged trocar incision or by lengthening two trocar incisions.

During the right adrenalectomy, the vena cava and right renal vein were identified, followed by the right central adrenal vein, which originates cranially on the lateral side of the inferior vena cava. The adrenal vein was then ligated with hem-o-lok clips and sectioned.

**Figure 2:**
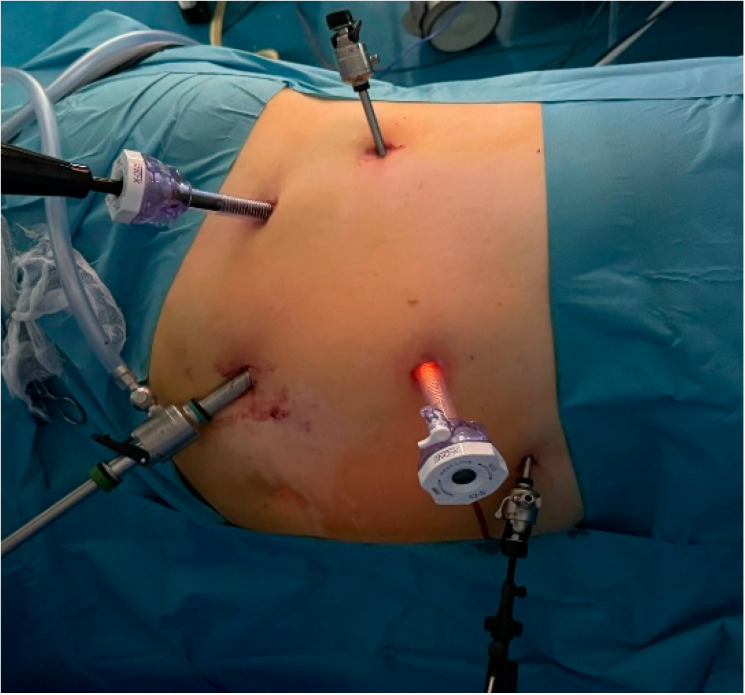
The positioning of the five trocars for the right adrenalectomy.

For the transperitoneal approach, the patient was positioned in lateral decubitus with the operating table tilted in an anti-Trendelenburg position to ensure a 45-degree horizontal angle. This positioning maximized the space between the iliac crest and the twelfth rib. The operative field was prepared to extend from the base of the xiphoid process down to the pubic symphysis, with the lateral boundary near the posterior axillary line. The primary surgeon and the assistant were situated in front of the patient, while the supporting team members were positioned on the opposite side, behind the patient. The Veress needle was inserted into the peritoneal cavity at the level of the umbilicus and connected to the CO_2_ insufflator to create a pneumoperitoneum. Once the working space was established at a pressure of 15 mmHg, the Veress needle was removed and the optical trocar was introduced. This enabled the inspection of the peritoneal cavity, followed by the insertion of additional trocars under direct visual guidance, positioned similarly to those used in a radical or partial nephrectomy. In the right adrenalectomy, mobilization of the liver was required, which was facilitated by the placement of an additional 5 mm trocar (Figure 2).

After incising the parietal peritoneum from the duodenum to the hepatic flexure of the colon to isolate the inferior vena cava, the central adrenal vein was identified and ligated using hemostatic clips. Ligation of the vein as the initial surgical step was crucial for reducing the release of secretory products during gland manipulation. Following this, the adrenal gland was dissected and the adrenal arteries were electrocoagulated. On the left side, the adrenal vein was identified after locating the left renal vein by sectioning the phrenocolic ligament and incising the parietal peritoneum. The adrenal vein was then ligated and divided, followed by dissection through an incision in the Gerota fascia, which allowed for the progressive identification and clipping of the arterial pedicles in the plane between the aorta and the gland. During the gland mobilization, the phrenic arterial pedicle and the superior adrenal artery at the level of the diaphragm could be identified. These were clipped and divided, followed by the specimen’s extraction in an organ retrieval bag.

For laparoscopic interventions where intraoperative ultrasound was performed, the Hitachi Arietta 60 ultrasound machine was utilized, equipped with a linear matrix LUS-type transducer that operating at a frequency of 7.0 MHz. The probe has a protective cover, making it easy to insert through a 12 mm trocar. To enhance the acoustic coupling, sterile saline solution or gel can be used. The transducer features an operational head with an adjustable working handle, enabling the deflection of the ultrasound beam to the right or left, similar to flexible endoscopes.

Laparoscopic intraoperative ultrasound was used in adrenal pathology to minimize the bleeding risk by quickly identifying glandular vascularization through Doppler velocimetry. Simultaneously, the degree of infiltration of the adrenal gland and surrounding tissues was assessed. Thanks to the benefits of intraoperative ultrasound, the dissection became faster and more precise, which led to a reduction in the operative time.

## 3. Results

A total of 128 patients were included in this study, categorized into four groups based on the surgical approach: IOUS-guided retroperitoneal (21.9%, *n* = 28), non-IOUS-guided retroperitoneal (39.1%, *n* = 50), IOUS-guided transperitoneal (23.4%, *n* = 30) and non-IOUS-guided transperitoneal (15.6%, *n* = 20).

The median age varied significantly across the groups (*p* = 0.040), where the IOUS-guided retroperitoneal group had the youngest median age (57 years, range: 45–67). The gender distribution was similar between the groups, with no significant differences (*p* = 0.811), although most of the patients were women. The patients with a higher BMI were more likely to be assessed using the IOUS-guided method, regardless of whether the approach was transperitoneal or retroperitoneal (*p* = 0.043). There was no significant difference in tumor location (left vs. right adrenal) between the groups (*p* = 0.884). However, larger tumors were more often addressed via the transperitoneal approach (Table 1). When categorized by tumor size, a higher proportion of tumors ≥ 6 cm was observed in the IOUS-guided transperitoneal group (63.3%), though this did not reach statistical significance (*p* = 0.072). Primary tumors were the most common in all the groups, where they ranged from 75% in the non-IOUS-guided transperitoneal group to 94.0% in the non-IOUS-guided retroperitoneal group. Secondary tumors were rare and evenly distributed across the groups.

Incidentalomas were more frequently observed in the IOUS-guided retroperitoneal group (57.1%) compared with the other groups (*p* = 0.022). Specific tumor subtypes, including adenomas, pheochromocytomas and cysts, showed no significant differences across the groups.

A power analysis was performed to evaluate whether this study had sufficient power to detect differences between the IOUS-guided and non-IOUS-guided groups. Using an independent samples *t*-test, power calculations were performed with an alpha level of 0.05 and a desired power of 0.80.

For a moderate effect size (Cohen’s d = 0.5), the required sample size per group was 56 participants. With the study’s actual sample sizes (*n* = 58 for the IOUS-guided group and *n* = 70 for the non-IOUS-guided group), this study had adequate power (>0.80) to detect moderate-to-large effects.

Secondary tumors primarily originated from pulmonary neoplasms (*n* = 7), followed by liver cancer (*n* = 3), malignant melanoma (*n* = 2) and retroperitoneal tumors (*n* = 3). The retroperitoneal approach demonstrated significantly better perioperative outcomes compared with the transperitoneal approach. Specifically, the retroperitoneal approach resulted in a shorter mean operative time and lower blood loss. The patients in the retroperitoneal group resumed oral intake earlier and had shorter hospital stays. Postoperative analgesia requirements were slightly lower in this group. Although intraoperative complications were more frequent in the retroperitoneal group, the difference was not statistically significant (Table 2).

**Figure 3:**
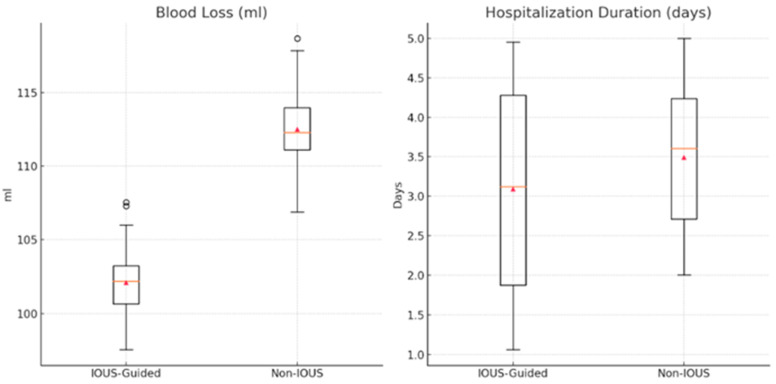
Comparison between IOUS-guided and non-IOUS-guided approach regarding blood loss and hospitalization duration.

When comparing the IOUS-guided and non-IOUS-guided techniques, several key differences were observed in the perioperative outcomes. The duration of the intervention was similar between the two groups. However, blood loss was significantly lower in the IOUS-guided group compared with the non-IOUS group (Table 3) (Figure 3). Additionally, the average duration of hospitalization was significantly shorter in the IOUS-guided group (*p*-value = 0.005).

In terms of intraoperative complications, none were reported in the IOUS-guided group, while five intraoperative complications were observed in the non-IOUS-guided group, according to the Clavien–Dindo classification. These complications included peritoneal breaches in the patients that underwent the retroperitoneal approach and minor liver damage during the transperitoneal approach, which was successfully managed with bipolar coagulation. However, this difference was not statistically significant (Table 3).

Overall, the patients that underwent an ultrasound-guided laparoscopic adrenalectomy experienced less intraoperative bleeding and fewer complications.

To reduce the selection bias and ensure a balanced comparison between the IOUS-guided and non-IOUS-guided groups, 1:1 nearest-neighbor propensity score matching was applied without replacement (Table 4). A caliper width of 0.2 of the standard deviation of the logit of the propensity score was used. The variables included in the matching process were age, BMI, tumor size (cm), laterality (left vs. right adrenal gland) and surgical approach (retroperitoneal vs. transperitoneal).

After propensity score matching, there were no significant differences between the IOUS-guided and non-IOUS-guided groups in terms of age (*p* = 0.742), BMI (*p* = 0.089), tumor size (*p* = 0.725), tumor laterality (*p* = 0.812), or surgical approach (*p* = 0.876). However, a trend toward significance was observed for BMI, though it did not reach statistical significance. The final matched cohort included 58 patients in each group, which ensured a well-balanced comparison for further statistical analyses (Table 4).

The regression analysis revealed that the IOUS guidance was significantly associated with a reduction in the hospitalization time (B = −0.8 days, 95% CI: −1.5 to −0.1, *p* = 0.025). Additionally, the retroperitoneal approach was independently associated with a greater reduction in the hospitalization time (B = −1.3 days, 95% CI: −2.1 to −0.6, *p* = 0.002). Other factors, such as age, BMI, tumor size and adrenal laterality, were not significantly associated with the hospitalization duration (*p* > 0.05) (Table 5).

For the intraoperative blood loss, IOUS guidance was associated with a significant reduction in the estimated blood loss (B = −40.2 mL, 95% CI: −72.4 to −8.0, *p* = 0.021). However, the retroperitoneal approach did not show a statistically significant effect on blood loss (B = −8.3 mL, 95% CI: −20.7 to 4.1, *p* = 0.119). Tumor size (*p* = 0.089) and BMI (*p* = 0.071) showed trends toward significance but did not reach statistical significance (Table 5).

## 4. Discussion

Laparoscopic ultrasound (IOUS) emerged later in the field of surgical ultrasound, mainly due to the requirement for specialized probes that could be inserted through conventional laparoscopic trocars. IOUS provides real-time imaging, enhancing the diagnosis and optimizing the treatment by compensating for the inability to palpate during laparoscopic procedures. It is a versatile, reproducible, safe, precise and fast method that is easy to use, making it an invaluable tool for surgeons. Currently, intraoperative ultrasound is used across a broad spectrum of surgical procedures, including pancreatic, hepatobiliary, neurological, cardiovascular, urological and endocrine surgeries [15,16,29,30,31,32,33,34].

In a laparoscopic adrenalectomy, IOUS plays a crucial role by significantly enhancing the precision and safety of this minimally invasive procedure, which is commonly performed for both benign and malignant pathologies. The utility of IOUS is particularly valuable for the precise localization of adrenal tumors, especially in cases involving small tumors or when anatomical changes or adhesions make identification challenging. IOUS helps ensure accurate removal and minimizes the risk of leaving tumor remnants, which is especially important during a partial adrenalectomy. The findings of this study highlight significant differences between the surgical approaches, where the retroperitoneal approach resulted in better outcomes, including shorter hospital stays and quicker resumption of feeding. However, this approach may have certain intraoperative disadvantages, as the complication rate seems to be higher. Although the slightly higher incidence of intraoperative complications did not reach statistical significance, it warrants further investigation. These findings are consistent with other significant studies in the literature that draw similar conclusions [35,36,37,38].

The use of imaging methods in surgery also enables real-time evaluation, which is valuable for differentiating solid from liquid formations, enhancing the staging process and improving the possibility of tumor resection. Additionally, the application of color Doppler imaging facilitates the identification of vascular structures, thereby reducing the bleeding time by enabling the faster ligation and sectioning of vascular elements. These advantages were reflected in statistically significant findings, as the patients who underwent ultrasound-guided procedures had much lower blood loss compared with those who did not. Intraoperative complications and postoperative analgesia requirements were not superior in ultrasound-guided cases. However, fewer intraoperative complications were noted when adjunctive imaging methods were employed. A larger patient cohort may provide stronger statistical significance for differences in hospitalization duration between the groups [33,39].

In our study, the primary impact on postoperative outcomes was observed in the retroperitoneal approach, which was associated with a reduced hospitalization time. Additionally, both the hospitalization duration and blood loss were significantly influenced by the use of intraoperative ultrasound (IOUS). However, the tumor size did not have a measurable impact on perioperative complications. This lack of correlation may be attributed to the fact that most cases in our cohort were incidentalomas and adenomas, which generally carry a lower risk of procedure-related complications, with potential risks being more related to endocrinological dysfunction rather than surgical factors.

Even though there was a linear trend of higher BMI in the IOUS-guided group, this association did not reach statistical significance. Given that a higher BMI and increased visceral fat could potentially complicate the procedure by leading to prolonged bleeding, our findings suggest that IOUS may play a role in mitigating such risks. Moreover, although the patients in the IOUS-guided group had a higher BMI, they experienced fewer complications, as observed in Table 5. These results highlight the potential benefit of IOUS in improving surgical safety, particularly in challenging cases, although further studies with larger cohorts are needed to confirm these findings.

In analyzing the literature, several authors explored the benefits of IOUS in a laparoscopic adrenalectomy, with important conclusions regarding its impact on the safety of the procedure. Heniford, one of the pioneers in laparoscopic adrenalectomy, emphasized the importance of using IOUS to identify tumors that are difficult to detect through other imaging methods or in cases involving small tumors. He also observed that IOUS reduces the risk of injury to neighboring structures by precisely guiding the resection [40]. Our regression analysis confirmed that IOUS guidance was independently associated with a significant reduction in blood loss, a critical factor in perioperative management. However, the lack of significant differences in operative time and postoperative analgesia suggests that IOUS primarily enhanced the safety rather than procedural efficiency.

Stephen E. Pautler and colleagues also emphasized the importance of IOUS in highlighting adrenal gland tumor formations during a laparoscopic partial adrenalectomy, especially when preoperative imaging does not provide precise information [41]. Other authors demonstrated that IOUS aids in characterizing adrenal gland lesions and accurately localizing them, further facilitating the dissection of the left adrenal vein with greater precision [42]. Laparoscopic adrenalectomy outcomes can be significantly improved with the use of IOUS, particularly in patients with a history of prior abdominal surgeries. This technique helps reduce complications, such as bleeding and injury to surrounding organs, contributing to safer procedures and better overall results [20].

Another valuable application of IOUS in laparoscopic adrenalectomy is assessing the feasibility of tumor resection, as highlighted by Golkowski in a 2007 article. This study presented a rare case of an oncocytoma in the adrenal cortex with extension and invasion into the inferior vena cava, as identified through IOUS. The final decision was to biopsy the lesions for patient safety reasons [43]. Additionally, IOUS is beneficial in patients with renal pathology, particularly when contrast-enhanced CT cannot be used. In such cases, contrast-enhanced ultrasound can be employed during surgery, as the contrast agent is eliminated via respiration, preserving kidney function while improving the imaging accuracy [44,45].

The younger median age observed in the IOUS-guided retroperitoneal group appeared as a trend in the regression analysis, where it potentially contributed to improved perioperative outcomes, as younger patients tend to have fewer comorbidities and faster recovery.

Although there is broad consensus on the benefits of IOUS in laparoscopic adrenalectomy, there is considerable debate regarding its necessity in all cases. Brunt et al. argued against its routine use, suggesting that advanced preoperative imaging techniques, such as MRI and CT, can adequately localize tumors. However, they acknowledge that IOUS remains valuable for selected patients [46].

The future of surgery increasingly revolves around robotic surgery, a method that has not been widely implemented due to high costs. Ultrasound-guided robotic procedures may become more common, offering advantages beyond laparoscopic surgery. Large-scale studies are needed to confirm these potential benefits definitively [6,47,48].

This study had several limitations, particularly the fact that it was not multicentric, the sample size was relatively small and the study design was retrospective.

## 5. Conclusions

This study showed that intraoperative ultrasound (IOUS) reduced the blood loss and shortened the hospital stay in laparoscopic adrenal surgeries. No intraoperative complications occurred in the IOUS-guided group, while three complications (peritoneal breaches and minor liver injuries) were observed in the non-IOUS group. The retroperitoneal approach combined with IOUS was associated with a shorter operative time, lower blood loss, faster oral intake resumption and reduced postoperative analgesia requirements. Overall, these findings support the integration of IOUS to enhance perioperative outcomes in laparoscopic adrenal surgery.

## Figures and Tables

**Table 1:** General characteristics of patients.

Variables	IOUS-Guided Retroperitoneal (*n* = 28)	Non-IOUS-Guided Retroperitoneal (*n* = 50)	IOUS-Guided Transperitoneal (*n* = 30)	Non-IOUS-Guided Transperitoneal (*n* = 20)	*p*-Value
Age (years)	57 (45–67)	62 (53–70)	63 (57–68)	61 (55–66)	0.040
Woman	16 (57.1%)	26 (52.0%)	18 (60.0%)	13 (65.0%)	0.811
Man	12 (42.9%)	24 (48.0%)	12 (40.0%)	7 (35.0%)	
BMI (kg/m^2^)	25.5 ± 3.4	24.2 ± 4.3	24.8 ± 3.9	23.9 ± 4.0	0.043
Left adrenal	16 (57.1%)	29 (58.0%)	24 (80.0%)	10 (50.0%)	0.884
Right adrenal	12 (42.9%)	21 (42.0%)	6 (20.0%)	10 (50.0%)	0.884
Average diameter (cm)	3.5 (3.0–6.0)	3.8 (4.0–7.0)	5.3 (4.2–8.9)	5.1 (3.0–8.5)	0.085
Tumor size					
<6 cm	19 (67.9%)	35 (70.0%)	11 (36.7%)	12 (60.0%)	0.072
≥6 cm	9 (32.1%)	15 (30.0%)	19 (63.3%)	8 (40.0%)	
Primary tumor	24 (85.7%)	47 (94.0%)	27 (90.0%)	15 (75.0%)	0.385
Secondary tumor	4 (14.3%)	3 (6.0%)	3 (10.0%)	5 (25.0%)	0.997
Incidentaloma	16 (57.1%)	12 (24.0%)	7 (23.3%)	7 (35.0%)	0.022
Cushing	2 (7.1%)	3 (6.0%)	3 (10.0%)	2 (10.0%)	1.000
Pheochromocytoma	3 (10.7%)	8 (16.0%)	3 (10.0%)	2 (10.0%)	0.901
Adenoma	15 (53.6%)	21 (42.0%)	12 (40.0%)	7 (35.0%)	0.373
Primitive carcinoma	2 (7.1%)	2 (4.0%)	4 (13.3%)	1 (5.0%)	0.998
Cyst	2 (7.1%)	5 (10.0%)	1 (3.3%)	3 (15.0%)	0.998

**Table 2:** Perioperative outcomes between retroperitoneal and transperitoneal surgical approaches.

Variable	Retroperitoneal (*n* = 78)	Transperitoneal (*n* = 50)	*p*-Value
Duration of intervention (mean ± SD, min)	85.9 ± 32.5	118.8 ± 49.6	<0.001
Blood loss (mean ± SD, mL)	60.8 ± 2.0	158.7 ± 6.1	<0.001
Resumption of food (mean ± SD, hours)	0.35 (0–2)	1.42 (1–4)	<0.001
Average hospitalization (mean ± SD, days)	3.4 (1–5)	4.6 (2–5)	<0.001
Postoperative analgesia (mean ± SD, hours)	2.3 (1–4)	2.9 (1–4)	<0.001
Intraoperative complications (*n*, %)	4 (5.1%)	2 (4.0%)	1.000

**Table 3:** Perioperative outcomes between IOUS-guided and non-IOUS-guided approaches.

Variable	IOUS-Guided (*n* = 58)	Non-IOUS (*n* = 70)	*p*-Value
Duration of intervention (mean ± SD, min)	98.4 ± 32.1	99.1 ± 30.8	0.917
Blood loss (mean ± SD, mL)	102.6 ± 1.9	112.3 ± 2.2	<0.001
Resumption of food (median, range, hours)	1.1 (1–3)	1.3 (1–3)	0.975
Hospitalization duration (median, range, days)	3.7 (1–5)	4.1 (2–5)	0.004
Postoperative analgesia (median, range, hours)	2.5 (1–5)	2.6 (1–5)	0.398
Intraoperative complications (*n*, %)	0 (0%)	5 (7.1%)	0.134

**Table 4:** Propensity score matching (1:1) for IOUS-guided vs. non-IOUS-guided groups.

Variable	IOUS-Guided (Matched) (*n* = 58)	Non-IOUS (Matched) (*n* = 58)	*p*-Value (Post-Matching)
Age (years, mean ± SD)	60.2 ± 8.5	59.8 ± 7.9	0.742
BMI (kg/m^2^, mean ± SD)	25.6 ± 3.8	24.9 ± 4.1	0.089
Tumor size (cm, mean ± SD)	4.1 ± 1.3	4.0 ± 1.4	0.725
Tumor laterality			
- Left adrenal (*n*, %)	40 (69.0%)	38 (65.5%)	0.812
- Right adrenal (*n*, %)	18 (31.0%)	20 (34.5%)	0.812
Surgical approach			
- Retroperitoneal (*n*, %)	34 (58.6%)	33 (56.9%)	0.876
- Transperitoneal (*n*, %)	24 (41.4%)	25 (43.1%)	0.876

**Table 5:** Multivariable linear regression analysis of factors influencing perioperative outcomes.

	Hospitalization (Days)			Blood Loss (mL)		
Variable	B-Coefficient	95% CI	*p*-Value	B-Coefficient	95% CI	*p*-Value
IOUS guidance	−0.8	(−1.5, −0.1)	0.025	−40.2	(−72.4, −8.0)	0.021
Retroperitoneal approach	−1.3	(−2.1, −0.6)	0.002	−8.3	(−20.7, 4.1)	0.119
Age (years)	0.05	(−0.08, 0.18)	0.265	2.1	(−0.3, 4.5)	0.087
BMI (kg/m^2^)	0.2	(−0.1, 0.5)	0.182	3.8	(−0.2, 7.8)	0.071
Tumor size (cm)	0.4	(−0.05, 0.85)	0.135	5.1	(−0.1, 10.3)	0.089
Adrenal laterality (left vs. right)	−0.2	(−0.9, 0.5)	0.610	−4.7	(−10.8, 1.4)	0.128

## Data Availability

The datasets generated and analyzed during the current study are not publicly available due to institutional restrictions but are available from the corresponding authors upon reasonable request.

## References

[1] Royer D.F., Rea P.M. (2019). Seeing with Sound: How Ultrasound Is Changing the Way We Look at Anatomy. Biomedical Visualisation.

[2] Kwon S.H., Gopal A.S. (2017). 3D and 4D Ultrasound: Current Progress and Future Perspectives. Curr. Cardiovasc. Imaging Rep..

[3] Pooh R.K., Maeda K., Kurjak A., Sen C., Ebrashy A., Adra A., Dayyabu A.L., Wataganara T., De Sá R.A.M., Stanojevic M. (2016). 3D/4D Sonography—Any Safety Problem. J. Perinat. Med..

[4] Makuuchi M., Torzilli G., Machi J. (1998). History of Intraoperative Ultrasound. Ultrasound Med. Biol..

[5] Bezzi M., Silecchia G., De Leo A., Carbone I., Pepino D., Rossi P. (1998). Laparoscopic and Intraoperative Ultrasound. Eur. J. Radiol..

[6] Pavone M., Seeliger B., Teodorico E., Goglia M., Taliento C., Bizzarri N., Lecointre L., Akladios C., Forgione A., Scambia G. (2024). Ultrasound-Guided Robotic Surgical Procedures: A Systematic Review. Surg. Endosc..

[7] Jiang Z., Salcudean S.E., Navab N. (2023). Robotic Ultrasound Imaging: State-of-the-Art and Future Perspectives. Med. Image Anal..

[8] Kane R.A. (2004). Intraoperative Ultrasonography: History, Current State of the Art, and Future Directions. J. Ultrasound Med..

[9] Marcal L.P. (2013). Intraoperative Abdominal Ultrasound in Oncologic Imaging. World J. Radiol..

[10] Di Mitri M., Thomas E., Di Carmine A., Manghi I., Cravano S.M., Bisanti C., Collautti E., Ruspi F., Cordola C., Vastano M. (2023). Intraoperative Ultrasound in Minimally Invasive Laparoscopic and Robotic Pediatric Surgery: Our Experiences and Literature Review. Children.

[11] Silas A.M., Kruskal J.B., Kane R.A. (2001). Intraoperative Ultrasound. Radiol. Clin. N. Am..

[12] Mihai I., Dura H., Teodoru C.A., Todor S.B., Ichim C., Grigore N., Mohor C.I., Mihetiu A., Oprinca G., Bacalbasa N. (2024). Intraoperative Ultrasound: Bridging the Gap between Laparoscopy and Surgical Precision during 3D Laparoscopic Partial Nephrectomies. Diagnostics.

[13] Alharbi F., Chahwan C., Le Gal S., Guleryuz K., Tillou X., Doerfler A. (2016). Intraoperative Ultrasound Control of Surgical Margins during Partial Nephrectomy. Urol. Ann..

[14] Bartoș A., Iancu I., Ciobanu L., Badea R., Spârchez Z., Bartoș D.M. (2020). Intraoperative Ultrasound in Liver and Pancreatic Surgery. Med. Ultrason..

[15] Lubner M.G., Mankowski Gettle L., Kim D.H., Ziemlewicz T.J., Dahiya N., Pickhardt P. (2021). Diagnostic and Procedural Intraoperative Ultrasound: Technique, Tips and Tricks for Optimizing Results. Br. J. Radiol..

[16] Chu K.-J., Kawaguchi Y., Hasegawa K. (2023). Current Use of Intraoperative Ultrasound in Modern Liver Surgery. Oncol. Transl. Med..

[17] Normahani P., Khan B., Sounderajah V., Poushpas S., Anwar M., Jaffer U. (2021). Applications of Intraoperative Duplex Ultrasound in Vascular Surgery: A Systematic Review. Ultrasound J..

[18] Ferrucci M., Milardi F., Passeri D., Mpungu L.F., Francavilla A., Cagol M., Saibene T., Michieletto S., Toffanin M., Del Bianco P. (2023). Intraoperative Ultrasound-Guided Conserving Surgery for Breast Cancer: No More Time for Blind Surgery. Ann. Surg. Oncol..

[19] Krishnamurthy V.D., Berber E., Shin J.J., Milas M., Mandel S.J., Langer J.E. (2017). Intraoperative Use of Ultrasound in Thyroid, Parathyroid, and Cervical Lymph Node Surgery. Advanced Thyroid and Parathyroid Ultrasound.

[20] Sebastian M., Rudnicki J. (2020). Recommendation for Laparoscopic Ultrasound Guided Laparoscopic Left Lateral Transabdominal Adrenalectomy. Gland Surg..

[21] Utsumi T., Iijima S., Sugizaki Y., Mori T., Somoto T., Kato S., Oka R., Endo T., Kamiya N., Suzuki H. (2023). Laparoscopic Adrenalectomy for Adrenal Tumors with Endocrine Activity: Perioperative Management Pathways for Reduced Complications and Improved Outcomes. Int. J. Urol..

[22] Di Buono G., Buscemi S., Lo Monte A.I., Geraci G., Sorce V., Citarrella R., Gulotta E., Palumbo V.D., Fazzotta S., Gulotta L. (2019). Laparoscopic Adrenalectomy: Preoperative Data, Surgical Technique and Clinical Outcomes. BMC Surg..

[23] Piramide F., Bravi C.A., Paciotti M., Sarchi L., Nocera L., Piro A., Lores M.P., Balestrazzi E., Mottaran A., Farinha R. (2023). Robot-Assisted Adrenalectomy: Step-by-Step Technique and Surgical Outcomes at a High-Volume Robotic Center. Asian J. Urol..

[24] Makay O., Erol V., Ozdemir M. (2019). Robotic Adrenalectomy. Gland Surg..

[25] Ndegwa R., Sywak M., Costin M. (2021). Intraoperative Ultrasound Facilitates Localization in a Morbidly Obese Patient Undergoing Posterior Retroperitoneoscopic Adrenalectomy. ANZ J. Surg..

[26] Gimm O., Duh Q.-Y. (2019). Challenges of Training in Adrenal Surgery. Gland Surg..

[27] Aporowicz M., Domosławski P., Czopnik P., Sutkowski K., Kaliszewski K. (2018). Perioperative Complications of Adrenalectomy—12 Years of Experience from a Single Center/Teaching Hospital and Literature Review. Arch. Med. Sci..

[28] Raffaelli M., De Crea C., Bellantone R. (2019). Laparoscopic Adrenalectomy. Gland Surg..

[29] Appleyard W., Meshaka R., Bebi C., Cho A., Chowdhury T., Smeulders N., Watson T. (2024). Intraoperative Ultrasound-Guided Paediatric Urological Surgery: A Pictorial Review. Pediatr. Radiol..

[30] Dong D., Ji Z., Li H., Yan W., Zhang Y. (2016). Laparoscopic Nephron Sparing Surgery Assisted with Laparoscopic Ultrasonography on Centrally Located Renal Tumor-Single Center Experience. Urol. Int..

[31] Schöppler G., Heinzelbecker J., Michaely H.J., Dinter D., Clevert D.-A., Pelzer A.E. (2012). Stellenwert des Ultraschalls in der Urologie. Urologe.

[32] Zhang G., Li Z., Si D., Shen L. (2017). Diagnostic Ability of Intraoperative Ultrasound for Identifying Tumor Residual in Glioma Surgery Operation. Oncotarget.

[33] Sopiński J., Kuzdak K. (2013). Navigation with Use of Intra-Operative Ultrasound in Search for Neoplastic Lesions of Endocrine Glands. Pol. J. Surg..

[34] Ćwik G., Solecki M., Wallner G. (2015). Applications of Intraoperative Ultrasound in the Treatment of Complicated Cases of Acute and Chronic Pancreatitis and Pancreatic Cancer-Own Experience. J. Ultrason..

[35] Ren T., Liu Y., Zhao X., Ni S., Zhang C., Guo C., Ren M. (2014). Transperitoneal Approach versus Retroperitoneal Approach: A Meta-Analysis of Laparoscopic Partial Nephrectomy for Renal Cell Carcinoma. PLoS ONE.

[36] Dong B., Zhan H., Luan T., Wang J. (2023). Laparoscopic Retroperitoneal Partial Nephrectomy for Cystic Renal Cell Carcinoma: A Video Vignette. Asian J. Surg..

[37] Fu J., Ye S., Ye H. (2015). Retroperitoneal Versus Transperitoneal Laparoscopic Partial Nephrectomy: A Systematic Review and Meta-Analysis. Chin. Med. Sci. J..

[38] Mihai I., Boicean A., Teodoru C.A., Grigore N., Iancu G.M., Dura H., Bratu D.G., Roman M.D., Mohor C.I., Todor S.B. (2023). Laparoscopic Adrenalectomy: Tailoring Approaches for the Optimal Resection of Adrenal Tumors. Diagnostics.

[39] Sadej P., Feld R.I., Frank A. (2009). Transplant Renal Vein Thrombosis: Role of Preoperative and Intraoperative Doppler Sonography. Am. J. Kidney Dis..

[40] Heniford B.T., Iannitti D.A., Hale J., Gagner M. (1997). The Role of Intraoperative Ultrasonography during Laparoscopic Adrenalectomy. Surgery.

[41] Pautler S.E., Choyke P.L., Pavlovich C.P., Daryanani K., Walther M.M. (2002). Intraoperative Ultrasound Aids in Dissection During Laparoscopic Partial Adrenalectomy. J. Urol..

[42] Lucas S.W., Spitz J.D., Arregui M.E. (1999). The Use of Intraoperative Ultrasound in Laparoscopic Adrenal Surgery: The Saint Vincent Experience. Surg. Endosc..

[43] Gołkowski F., Buziak-Bereza M., Huszno B., Bałdys-Waligórska A., Stefańska A., Budzyński A., Okoń K., Chrzan R., Urbanik A. (2007). The Unique Case of Adrenocortical Malignant and Functioning Oncocytic Tumour. Exp. Clin. Endocrinol. Diabetes.

[44] Le O., Wood C., Vikram R., Patnana M., Bhosale P., Bassett R., Bedi D. (2017). Feasibility of Contrast-Enhanced Intraoperative Ultrasound for Detection and Characterization of Renal Mass Undergoing Open Partial Nephrectomy. J. Ultrasound Med..

[45] Cagini L., Gravante S., Malaspina C.M., Cesarano E., Giganti M., Rebonato A., Fonio P., Scialpi M. (2013). Contrast Enhanced Ultrasound (CEUS) in Blunt Abdominal Trauma. Crit. Ultrasound J..

[46] Brunt L.M., Bennett H.F., Teefey S.A., Moley J.F., Middleton W.D. (1999). Laparoscopic Ultrasound Imaging of Adrenal Tumors during Laparoscopic Adrenalectomy. Am. J. Surg..

[47] Ciocan R.A., Graur F., Ciocan A., Cismaru C.A., Pintilie S.R., Berindan-Neagoe I., Hajjar N.A., Gherman C.D. (2023). Robot-Guided Ultrasonography in Surgical Interventions. Diagnostics.

[48] Bai L., Zhao L., Ren H. (2025). Ultrasound Guidance and Robotic Procedures: Actual and Future Intelligence. Handbook of Robotic Surgery.

